# The Unequal Effect of the COVID-19 Pandemic on the Labour Market and Income Inequality in China: A Multisectoral CGE Model Analysis Coupled with a Micro-Simulation Approach

**DOI:** 10.3390/ijerph19031320

**Published:** 2022-01-25

**Authors:** Qi Zhang, Xinxin Zhang, Qi Cui, Weining Cao, Ling He, Yexin Zhou, Xiaofan Li, Yunpeng Fan

**Affiliations:** 1 School of Economics and Resource Management, Beijing Normal University, Beijing 100875, China; 03035@bnu.edu.cn (Q.Z.); xinxinzhang@mail.bnu.edu.cn (X.Z.); 202021410018@mail.bnu.edu.cn (W.C.); 201831410010@mail.bnu.edu.cn (L.H.); 202021410006@mail.bnu.edu.cn (X.L.); 2 China Institute for Poverty Reduction, Beijing Normal University, Beijing 100875, China; 3 China Rural Revitalization and Development Research Center, Beijing Normal University, Zhuhai 519087, China; 4 Beijing Key Lab of Study on Sci-Tech Strategy for Urban Green Development, Beijing Normal University, Beijing 100875, China; 5 Center for Innovation and Development Studies, Beijing Normal University, Zhuhai 519087, China; 6 Institute of Finance & Banking, Chinese Academy of Social Sciences, Beijing 100710, China; fypifb@cass.org.cn

**Keywords:** COVID-19 pandemic, labour market, CGE model, the micro-simulation approach, income inequality

## Abstract

The COVID-19 pandemic had an unequal impact on the employment and earnings of different labourers, consequently affecting households’ per capita income and income inequality. Combining a multisector computable general equilibrium model of China with a micro-simulation approach, this study aims to analyse the unequal effect of the COVID-19 pandemic on China’s labour market and income inequality. The results confirm the unequal impact of the pandemic on the employment and earnings of different labourer types. Labourers who are female, live in urban areas, and have relatively low education levels would suffer greater losses in employment and earnings. The pandemic would reduce household per capita income by 8.75% for rural residents and 6.13% for urban residents. While the pandemic would have a larger negative impact on the employment and earnings of urban labourers, it would have a greater negative impact on the household per capita income of rural residents. Moreover, the per capita income of low-income households is more vulnerable to the pandemic, and the number of residents living below the poverty line would increase significantly. Thus, the pandemic would aggravate income inequality in China and threaten the livelihoods of poor families. This study could inform researchers exploring the distributional effect of the COVID-19 pandemic in developing countries.

## 1. Introduction

As one of the most significant global public health crises in recent decades, the COVID-19 pandemic posed severe threats to residential health and economy. Strict social distancing and massive shutdown of economic activities during the pandemic reduced enterprises’ production and decreased consumer expenditure, inflicting a severe shock to China’s economy [1,2,3]. In 2020, China’s GDP grew by only 2.3% [4], much lower than the projected growth rate, assuming no pandemic (6.0% [5]). The pandemic also triggered considerable shocks to China’s labour market. Numerous labourers lost their jobs, and the unemployment rate in urban areas reached 4.2% in 2020, 0.6 percentage points higher than that in 2019 [4]. The annual salary in non-private enterprises grew by 5.2% in 2020, the lowest since 1984, while in private enterprises, it grew by 5.3%, the second lowest since 2009. Consequently, residents’ per capita income grew by only 2.1% in 2020, 3.7 percentage points lower than in 2019. 

Deduced from previous studies, the COVID-19 pandemic is expected to have an unequal impact on the labour market and aggravate income inequality. Fading economic activities in the producing sectors have reduced the demand for labour and lowered wages, especially in sectors directly exposed to the pandemic, such as construction, education, hotels, and restaurants [6,7,8,9]. Owing to the employment structure of different sectors, the COVID-19 pandemic may have an unequal impact on the employment and earnings of different labourers. Several studies have found that labourers who are older, have a low level of education, non-regular employees, and female would suffer greater losses [10,11,12,13,14,15,16]. Thus, the first research hypothesis of the study is that the COVID-19 pandemic would have an unequal impact on the labour market. Furthermore, through influencing the labour market, the COVID-19 pandemic may have a differing impact on the income of different households. As the employment and earnings of labourers with low education levels are more significantly reduced, the pandemic would have a greater negative impact on the income of low-income households than their more advantaged counterparts [17,18,19]. Strict disease control measures hindered migrant workers from returning to work, reduced the income of rural households, and aggravated the income disparity between rural and urban households [8,20,21]. Hence, the second hypothesis is that income inequality would be aggravated by the COVID-19 pandemic. Although several studies have preliminarily analysed the distributional effects of the pandemic in developed countries [10,12,20], the unequal impact of the pandemic on China’s labour market and income inequality has been scarcely explored.

Combining a multisector computable general equilibrium (CGE) model with a micro-simulation approach, this study aims to assess the unequal impact of the COVID-19 pandemic on China’s labour market and income inequality. This study fills the gaps in the literature in three aspects. First, it quantitatively evaluates the unequal impact of the COVID-19 pandemic on the employment and earnings of different types of labour through the CHINAGEM model. Second, it analyses the unequal impact of the COVID-19 pandemic on the per capita income of households with different income levels through the combination of a CGE model and a micro-simulation approach. Third, to the best of our knowledge, this study is the first to quantitively examine the impact of the COVID-19 pandemic on income inequality between/among urban and rural residents in China. Moreover, several policy implications are provided for countries to mitigate the adverse impact of the COVID-19 pandemic on income inequality. This study is valuable to researchers exploring the distributional effect of the COVID-19 pandemic in developing countries.

The paper is structured as follows: Section 2 reviews the relevant literature; Section 3 introduces the CHINAGEM model and the micro-simulation approach; Section 4 shows the simulation results for the unequal effect of the COVID-19 pandemic on China’s labour market and household income; Section 5 discusses the results and concludes the study with several policy implications.

## 2. Literature Review

Several studies have estimated the impact of the COVID-19 pandemic on the labour market [6,7,8,9,22,23,24,25]. The fading economic activities of the producing sectors reduced the demand for labour and lowered wages, especially in sectors directly exposed to the pandemic. Further, transportation, housing, and other constraints hindered migrant workers from returning to work [8,9]. Fang et al. [24] found that the number of newly posted jobs released in the largest online platform in China within the first 13 weeks after the Wuhan lockdown (from 23 January 2020) was one-third lower than that of the same weeks in 2018 and 2019. Cai, Zhang, and Liu [25] conducted a nationwide survey and found that the unemployment rate reached a high level of 11% by June 2020. For the entire 2020, Cui et al. [26], using a CGE model, projected a 2.72% reduction in total employment. Zhang [22] found that by the end of November 2020, about 4.4% of 2019 incumbent workers were still unemployed from an employee-tracking survey in China. Moreover, several sectors, including construction, education, hotels, and restaurants, had much more significant employment losses, as they were directly exposed to the pandemic [27,28,29,30].

An increasing number of studies explored the unequal impact of the COVID-19 pandemic on the employment and earnings of different labourers [10,11,12,13,14,15,16,31,32,33,34]. Hoshi et al. [31] found that unemployment was more severe for the non-regular workers, those with low education levels, females, and those aged 31 to 45 years in Japan. Mongey, Pilossoph, and Weinberg [10] suggested that workers who are less educated and have lower incomes and fewer liquid assets were more economically vulnerable to the pandemic in the United States. Hoehn-Velasco, Silverio-Murillo, and Miyar [11] found that the most affected labourers included the youngest, oldest, low-income earners, those hired by small- and medium-sized firms, and those engaged in hospitality-focused services. Furthermore, several studies have suggested that female labourers were more significantly affected by the pandemic than their male counterparts [12,13,14,15]. For example, Albanesi and Kim [15] found that female workers, especially those that have born children, experienced a more substantial employment reduction than male workers. Graeber, Kritikos, and Seebauer [12] also suggested that female labourers were about one-third more likely to experience income losses than their male counterparts. As for China, previous studies have scarcely analysed the unequal impact of the COVID-19 pandemic on the employment and earnings of labourers with different features. The only exception is Che, Du, and Chan [16], who suggested that migrant rural workers in China who are less educated and less skilled had a higher rate of unemployment. 

By affecting the per capita income of different households, the COVID-19 pandemic likely increased income inequality in China. On the one hand, rural residents may suffer greater income losses relative to urban residents, as rural workers are less employed on a salary basis, and migrant workers from rural areas are confined to their villages and unable to return to work in cities [8,20]. The sudden reduction or halt in remittance flows from migrant workers would further reduce the income of rural households [21,35]. On the other hand, as the pandemic has a more significant negative impact on the low-income households than their advantaged counterparts, it may exacerbate income inequality [17,18,19,36,37,38]. Almeida et al. [19] found that the COVID-19 pandemic was likely to significantly affect households’ disposable income in the European Union, with lower-income households being more severely hit. Delaporte, Escobar, and Pea [37] found that the pandemic would deteriorate the income of low-income households more severely and push numerous households into poverty. A survey conducted by Luo et al. [38] demonstrated that 7.1% of rural households in China would possibly fall into poverty due to the pandemic, and 23% who had escaped poverty were likely to fall back. 

As it is challenging to incorporate a large number of representative households into the traditional CGE models, several studies have used an integrated methodology, combining the CGE models and the micro-simulation approaches, to analyse changes in household incomes caused by exogenous shocks [39,40,41,42]. Based on the simulation results of the CGE models, the micro-simulation approach can calculate the changes in income of numerous households with different features [43]. Wang et al. [40] used a recursive dynamic CGE model with a top-down behavioural micro-simulation approach and indicated that population ageing negatively impacts China’s poverty reduction but benefits income equality. Heyndrickx, Vanheukelom, and Proost [42] combined a regional CGE model and a household micro-simulation approach to assess the distributional effects of transport tax reform. van Ruijven, O’ Neill, and Chateau [44] used a global CGE model and a top-down micro-simulation approach to analyse the consequences of climate change and climate policy on different household types (based on their income level, expenditure pattern, and other socioeconomic characteristics). These studies have illustrated the effectiveness of the micro-simulation approach in examining the impacts of exogenous shocks on household income and income inequality.

Previous studies have contributed to the understanding of the pandemic impact on the labour market and household income and explored changes in income inequality preliminarily. However, most of them have focused on the pandemic impact on the labour market and household income in developed countries. The unequal impact of the COVID-19 pandemic on China’s labour market has not been investigated comprehensively. Furthermore, existing literature shows that rural residents may suffer greater income losses relative to urban ones, exacerbating the income disparity between urban and rural residents. Previous studies did not quantitatively probe the impact of the COVID-19 pandemic on income inequality between/among urban and rural residents in China. 

## 3. Methodology

**Figure 1:**
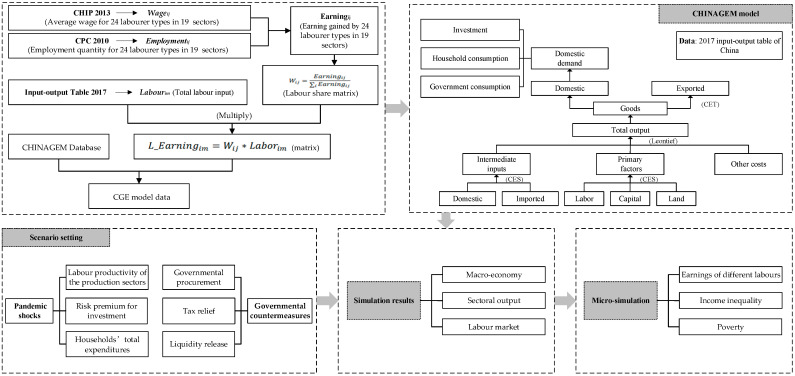
The flow chart of this study. Source: the authors.

As shown in Figure 1, this study combines a multisector CGE model with a micro-simulation approach and assesses the unequal impact of the COVID-19 pandemic on the labour market and income inequality in China.

### 3.1. CHINAGEM Model

**Figure 2:**
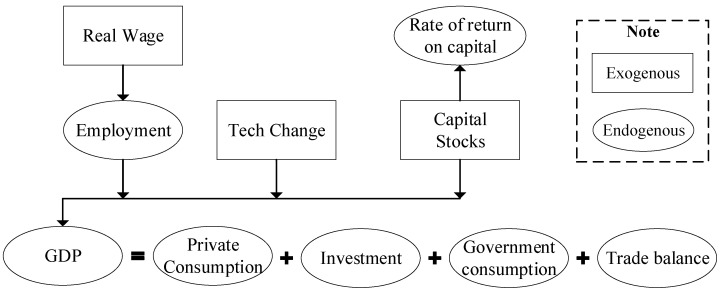
The theoretical framework of the CHINAGEM model. Source: Cui et al. [26].

Several studies have used CGE models to analyse the economic impacts of infectious diseases [26,45,46]. Following them, this study employs a multisectoral CGE model, CHINAGEM, to evaluate the effects of the COVID-19 pandemic on the employment and earnings of different labourers in China. The model was developed by the Institute of Science and Development, Chinese Academy of Sciences, and Centre of Policy Studies, Victoria University of Australia [47,48]. The CHINAGEM model is a comparative static analysis model, assuming that the market is perfectly competitive and there are constant returns to scale. All product and input markets are cleared so that the equilibrium of total supply and demand determines endogenous variables, such as price, wages, and land rent. There are six economic agents (production, investment, consumption, government, foreign, and inventory) and three primary factors (labour, capital, and land). The theoretical framework of the CHINAGEM model is shown in Figure 2. For detailed formulas, refer to Cui et al. [49] and Cui et al. [26].

**Figure 3:**
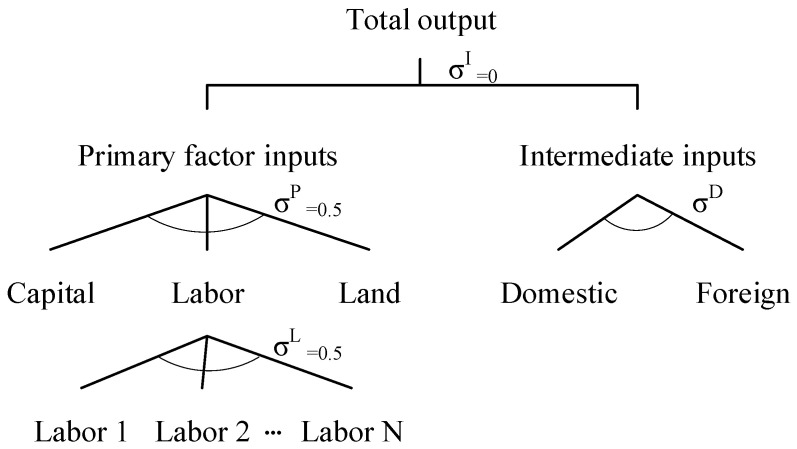
The nesting structure of the production inputs of sectors. Source: the authors.

To save space, this study briefly introduces the nesting structure of the inputs for the producing sectors (Figure 3). On the top level, the total input of each producing sector is composed of the primary factor and intermediate inputs, which is depicted by a Leontief function, assuming that their utilisation is fixed proportionally to the total input. On the next level, the primary factor input consists of labour, capital, and land, as depicted by the constant elasticity of substitution (CES) function with a substitution elasticity of 0.5. The intermediate goods include domestic and foreign goods according to the Armington assumption, suggesting that domestically produced goods and foreign goods have incomplete substitution. On the bottom, following Mu et al. [50], the labour input of each producing sector is split into 24 types along with three properties of labourers. These properties include the living areas (urban or rural), gender (male or female), and education levels (elementary school, middle school, high school, junior college, regular college, and postgraduate). By combining the three properties, 24 labourer types are obtained (2 × 2 × 6 = 24). A CES function is employed to describe the substitution between different types of labourers with an elasticity of 0.5.

To establish the database of the CHINAGEM model, this study uses China’s recently published input–output table with the base year of 2017 [51], which provides the total labour input of 149 producing sectors. Then, the total labour input of each sector is split into 24 labourer types based on two datasets. First, the 6th Chinese Population Census (CPC 2010), conducted in 2010, provided the employment quantity for 24 labourer types in 19 aggregated sectors. Second, the Chinese Household Income Project (CHIP 2013) database, a household survey conducted in 2013 with a sample of 58,743 individuals in 17,146 households, provided individuals’ wages. As the CPC 2010 only has 19 aggregated sectors (Table A1), the average wage of each labourer type (*Wage_ij_*) is calculated for these aggregated sectors based on the CHIP 2013 database. The earnings *(Earning_ij_*) gained by 24 labourer types from 19 aggregated sectors are obtained by multiplying the employment quantity (*Employment_ij_*) with the average wage.

(1) $$Earning_{ij}=Wage_{ij}*Employment_{ij}$$

In Equation (1), *i* indexes the labourer type, and *j* indexes the aggregated sector. For each of the 19 aggregated sectors, the weight of the earnings is gained by each labourer type from the sector’s total labour input (*W_ij_*).

(2) $$W_{ij}=\frac{Earning_{ij}}{\sum_{i}Earning_{ij}}$$

By mapping the 19 aggregated sectors of the CPC 2010 to 149 sectors of the 2017 input-output table (see Appendix A), the earnings gained by each labourer type is calculated by multiplying the weight matrix (*W_ij_*) with the total labour input (*Labour_im_*) of the mapped subdivided sectors *m*. The *m* indexes the sector from the 2017 input–output table that is mapped with the aggregated sector *j*. Finally, a matrix is obtained for the earnings gained by 24 labourer types from the 149 sectors (*L_Earning_im_*).

(3) $$L\_Earning_{im}=W_{ij}*Labour_{im}$$

As for the closure of the simulation, this study, consistent with previous studies, analyses the damages caused by the COVID-19 pandemic in the short term. Capital is exogenous in producing sectors with a sectoral-differentiated rate of return (Figure 2). The real wage is unchanged while allowing for unemployment. Tax rates and technology are determined exogenously.

### 3.2. The Micro-Simulation Approach

Although the CHINAGEM model could simulate the unequal impact of the COVID-19 pandemic on employment and earnings for different labourer types, it is difficult to reveal the effects on household income and the income equality. Household income is from the factor rents (including labour and capital) and transfer income from both government and non-government institutions. The CGE models could simulate the changes in the employment and income of labourers; however, they could not directly estimate the income changes of households with different features. Several studies have coupled the CGE models with the micro-simulation approach, transferring the simulated macroeconomic results to the microdata covering numerous households to simulate the impact of policies on household income and the income equality [40,52,53]. Following these studies, the CHINAGEM model is combined with a micro-simulation approach to simulate the impact of the COVID-19 pandemic on household income and the income inequality in China. 

As reviewed by Debowicz [53], previous studies developed two methods to link CGE models with the microdata, including the integrated link and layered link. The former integrates the microdata into the CGE model and works well when there are small groups in the models. However, within-group changes in income distribution cannot be considered because the households in the same group are assumed to be unified. The latter solves this problem and contains the behaviour-layered link and non-behaviour-layered link methods depending on whether all the households in a group are affected in the same way by changes in the macro variables [40]. The behaviour-layered link method is further divided into the top-down micro-simulation [43,53] and the bottom-up micro-simulation [52]. Following Wang et al. [40], this study employs the non-behaviour-layered method for the top-down micro-simulation.

The total income of each household is defined in Equation (4):

(4) $$YH^{0}=\frac{1}{N}\left(\underset{i}{\sum }L\_Earning_{im}^{0}*D_{i}+Y_{NL}^{0}\right)$$

where *YH* is the total income of each household, which is the summation of labour income and non-labour income; *N* is the number of family members within a household. For the former, the household’s labour income is the summation of the earnings gained by family members. *D_i_* is a dummy variable representing the family member’s work status: it equals 1 if the member has a job; otherwise, it is 0. The non-labour income of the household, *Y_NL_*^0^, includes capital income and the income transferred from government and non-government institutions. 

The CHIP 2013 database provides data on the labour income of family members and non-labour income for each household, used to calculate the indicators measuring income inequality, such as the Gini coefficient and Lorenz curve. It is regarded as a benchmark for household income without the COVID-19 pandemic. The CGE model provides the impact of the pandemic on the earnings gained by 24 labourer types from 149 producing sectors. Following Wang et al. [40], the labour incomes of family members are linked with the changes in earnings by labourer type and industry via the top-down non-behaviour-layered link (Equation (5)) and calculate the labour income of family members considering the effects of the pandemic.

(5) $$L\_Earning_{im}^{1}=L\_Earning_{im}^{0}*\left(1+{\hat{l\_earning}}_{im}\right)$$

where ${\hat{l\_earning}}_{m}$ is the percentage change in earnings earned by the labourer type *i* from industry *m*, estimated by the CHINAGEM model. As the changes in households’ non-labour income could not be estimated from the CGE model, this study assumes that the non-labour income remains unchanged. Household income under the COVID-19 pandemic can then be calculated (Equation (6)). Then, the income inequality indicators are also calculated, including the Gini coefficient and Lorenz curve. Comparing household income and the income inequality indicators with and without the pandemic, this study can assess the impact of the COVID-19 pandemic on household income and income inequality.

(6) $$YH^{1}=\frac{1}{N}\left(\underset{i}{\sum }L\_Earning_{im}^{1}*D_{i}+Y_{NL}^{0}\right)$$

### 3.3. Scenario Setting

To establish the scenarios of the COVID pandemic, previous studies considered the multiple shocks of the COVID-19 pandemic to the economy, including the reduction of labour productivity, the loss of labour forces, the increase in investment premium, the decrease in household consumption, and the increase in protective costs [26]. Further, some studies incorporated governmental countermeasures into the scenario design [54]. Following these studies, this study establishes a scenario for the pandemic considering three types of economic shocks and three types of governmental countermeasures and quantifies the shocks based on the statistical data of 2020 in China as follows: 

(1) The labour productivity of the production sectors declined by 5.60%. The nationwide extension of the spring festival vacation, insufficient operation, and strict social distancing hindered the spread of the pandemic effectively but also lowered the labour productivity of the production sectors. The loss of workdays is calculated as the negative shock to the labour productivity of the producing sectors. As estimated by Zheng et al. [55], labourers’ workdays in 2020 fell by 5.60% in China. 

(2) The risk premium for investment increased by 1.16%. The pandemic posed a significant risk to investment, which significantly increased the costs of investment. As a higher risk premium of investment is required, following McKibbin and Fernando [54], this study assumes that the investment risk premium in China increased by 1.16%. 

(3) Households’ total expenditures dropped by 4.83% [4]. Influenced by the pandemic, households significantly reduced their consumption expenditure, especially on accommodation and food services, textiles, clothes, and transport equipment. In addition to the negative impact on household expenditure, the pandemic had a different impact on consumer goods. The shocks are obtained for the consumption of food (3.92%), clothing and shoes (−8.48%), retail (−3.63%), transportation (−4.20%), accommodation (2.57%), food service (−17.03%), and culture and entertainment (−19.74%) in 2020.

(4) Three types of governmental countermeasures are considered, such as the governmental procurement, tax relief, and liquidity release. Following Zhou et al. [56], the shocks on these countermeasures are quantified. China’s government increased its spending on clinical and health services by 4.0% and social welfare by 8.2% in 2020. Simultaneously, the government reduced the value-added tax levied on the pandemic prevention and control materials by 8–48%. The government also released liquidity in the capital market to stimulate the economy by reducing the bidding rate of 7-day and 14-day reverse repurchases in the open market by 2.40% and 2.55%, respectively. This consequently reduced the financing cost of firms by approximately 4%.

## 4. Results

### 4.1. The Impact on China’s Macro-Economy

**Figure 4:**
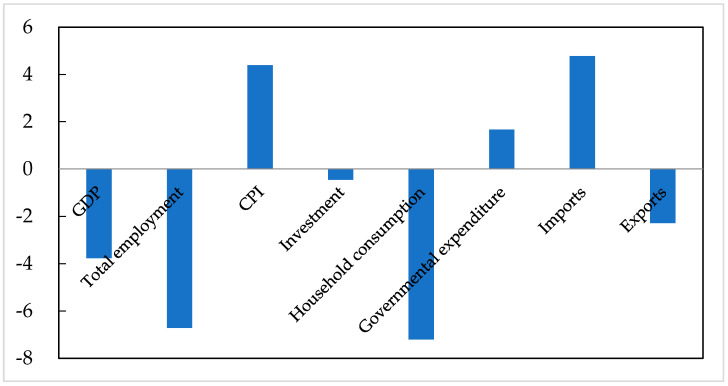
The impact of the COVID-19 pandemic on China’s macro-economic variables (%). Source: CHINAGEM model.

Consistent with expectations, the COVID-19 pandemic severely hurt China’s macro-economy (Figure 4). The pandemic lowered industrial output and triggered unemployment by reducing sectorial labour productivity, total investment, and household consumption, thereby negatively affecting China’s GDP. Although governmental countermeasures could mitigate economic damages, they could not buffer the economic losses completely. The national GDP is projected to decrease by 3.77%. Compared with the IMF’s projection for China’s GDP in 2020 before the pandemic (6% [5]), our simulation result indicates that China’s GDP would grow by 2.23% in 2020, which is much near to the official statistics (2.3%). Further, the pandemic would reduce total investment and household consumption by 0.45% and 7.21%, respectively. Due to the increased expenditure on health and social security, the governmental expenditure would rise by 1.66%. Meanwhile, the pandemic would raise imports by 4.77% and reduce exports by 2.28%. Total employment would fall by 6.72%. In addition, the pandemic would raise the consumer price index (CPI) by 4.39%. 

**Figure 5:**
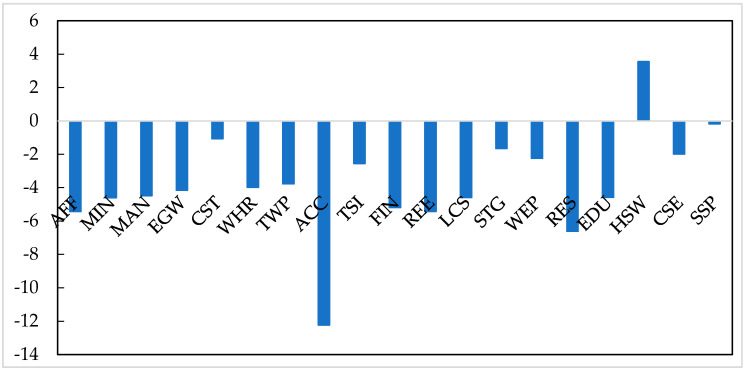
The impact of the COVID-19 pandemic on the output value of aggregated sectors (%). Source: CHINAGEM model.

To show the simulation results clearly, the output changes of 149 original sectors are aggregated to 19 aggregated sectors weighted by sectorial output values. The COVID-19 pandemic has a far-reaching, unequal impact on the output value of producing sectors. The output value of the producing sectors would decline by an average of 3.75% (Figure 5). Among them, accommodation and food services (ACC) would have the largest output reduction (−12.23%). Following the ACC, the output value of the residential services (RES), real estate (REE), finance (FIN), and agriculture (AFF) would also be largely damaged (fall by over 5%). Compared with these sectors, the pandemic would slightly reduce the output value of public administration (SSP), construction (CST), and scientific research and development (STG) by less than 2%. Furthermore, the output value of health and social welfare (HSW) is positively affected by the pandemic, as the government expanded its expenditure on clinical and health services.

### 4.2. The Impact on Labourer Employment and Earnings of Different Sectors

**Figure 6:**
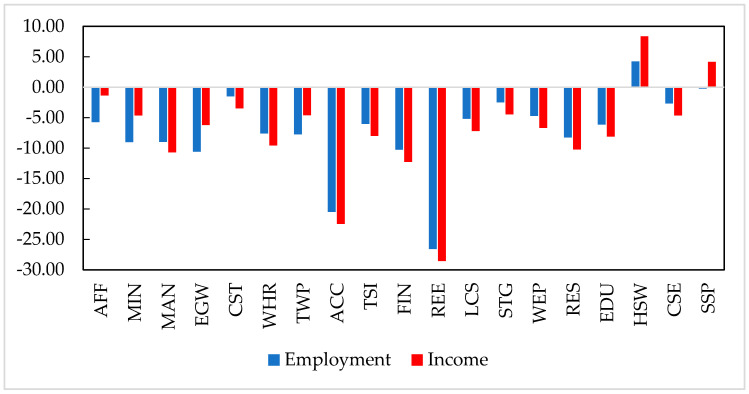
The impact of COVID-19 pandemic on labourer employment and earnings of sectors (%). Source: CHINAGEM model.

The COVID-19 pandemic would negatively affect labourer employment and earnings for most producing sectors, which are highly consistent with output losses of the sectors (Figure 6). The pandemic would reduce the output value of producing sectors and cut down their demand for labour input, resulting in a decrease in labourer employment and earnings. Furthermore, sectors with larger output losses would have a greater reduction in employment and earnings. For example, real estate (REE) and accommodation and food services (ACC) would have the largest decreases in employment, falling by 26.27% and 20.47%, respectively. On the one hand, the pandemic would damage the output value of these sectors, directly reducing their demand for labour input. On the other hand, these sectors are labour-intensive, as labour accounts for a high proportion of their production costs. Hence, the employment of these sectors would decline significantly. Accordingly, the earnings gained by labourers from the REE and ACC would decline by 28.55% and 22.45%, respectively. Following the REE and ACC sectors, the employment of energy and water supply (EGW) and finance (FIN) would decline by 10.58% and 10.27%, respectively. The output decreases of these sectors would also reduce their demand for labour input, resulting in a significant reduction in employment. Accompanied with employment decreases, the earnings gained by labourers from EGW and FIN would fall by 6.19% and 12.25%, respectively. Hence, the pandemic would significantly reduce the employment and earnings of labourers for most producing sectors. 

This study also finds that the COVID-19 pandemic would have a relatively lower impact on labourer employment and earnings in several sectors, including public administration (SSP), construction (CST), scientific research and development (STG), and culture and entertainment (CSE). As the pandemic has a relatively slighter impact on the outputs of these sectors, their demand for labour input would also decline slightly. The labourer employment of these sectors would decrease by less than 3%, accompanied by a decrease in earnings of less than 5%. In addition, the labourer employment in health and social welfare (HSW) would increase by 4.36% because the government expanded the expenditure on health and clinical services, which consequently increased the demand for labour input of the HSW. The earnings gained by labourers from the HSW would rise by 8.37%.

### 4.3. The Impact on the Employment and Earnings of Different Labourers

The unequal impact of the COVID-19 pandemic on the employment and earnings of different labourer types are also examined. Owing to the employment structure of different sectors, the pandemic would have heterogeneous effects on the employment and earnings of different labourer types (Table 1).

First, labourers in urban areas would suffer larger damages from the COVID-19 pandemic to their employment and earnings than their counterparts in rural areas. The employment of labourers in urban areas would decline by an average of 6.60%, accompanied by an average decrease of 7.03% in earnings. Meanwhile, the employment and earnings of labourers in rural areas would fall by an average of 6.16% and 5.44%, respectively. This could be mainly explained by two factors. First, urban areas hosted a larger proportion of producing sectors largely damaged by the pandemic, such as the ACC, RES, and REE. Second, urban labourers accounted for a larger proportion of the labour input in these sectors. In contrast, the output of sectors that are more likely to hire rural labourers, such as the AFF and CST, are relatively slightly damaged by the pandemic. This finding may be attributable to other factors that are not depicted by the CHINAGEM model. For example, the population living in an urban area has a higher risk of severe infection (hospitalisation or mortality) [57].

Second, female labourers would suffer slightly larger damages to their employment and earnings than male labourers, which is consistent with previous studies [12,13,14]. The employment of female labourers would decline by an average of 6.55%, accompanied by an average decrease of 6.42% in earnings. Meanwhile, the employment and earnings of male labourers would fall by an average of 6.21% and 6.05%, respectively. While the male is at a higher risk of severe infection [57], our findings are evidenced by the fact that female labourers accounted for a larger proportion of labour input in the primarily affected sectors, such as the ACC and RES. 

Third, labourers with relatively low education levels would experience significantly larger decreases in employment and earnings than their counterparts with high education levels. In urban areas, labourers with education levels below high school would suffer employment and earnings losses by over 7% for both male and female labourers. Meanwhile, labourers with education levels above junior college would suffer much smaller losses in their employment and earnings. Similarly, in rural areas, labourers with education levels below high school would have a decrease in employment and earnings of over 6%, much larger than those with education levels above junior college. 

To conclude, labourers who are the female, live in urban areas, and have low education levels would suffer the largest losses in their employment and earnings. Comparably, labourers with high education levels would experience smaller decreases in their employment and earnings, regardless of their living regions and gender.

### 4.4. The Impact on Household Income and the Income Inequality

**Figure 7:**
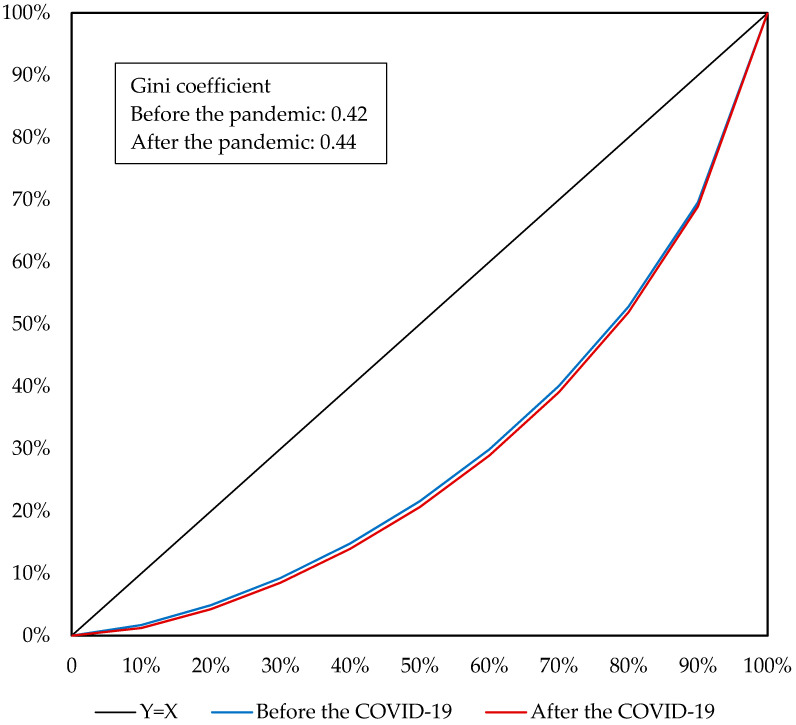
Lorentz curve of all residents before and after the COVID-19 pandemic. Source: Author’s calculation.

**Figure 8:**
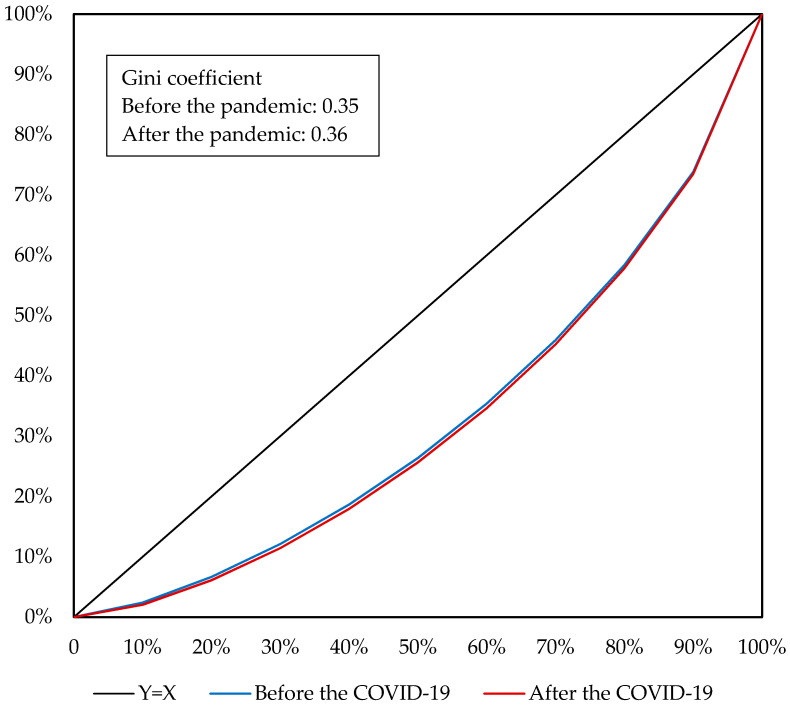
Lorentz curve of urban residents before and after the COVID-19 pandemic. Source: Author’s calculation.

**Figure 9:**
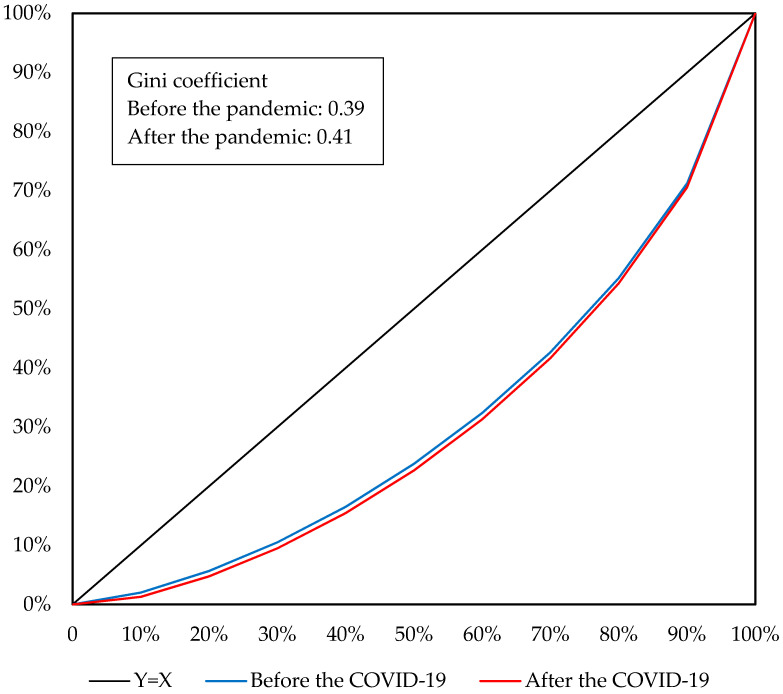
Lorentz curve of rural residents before and after the COVID-19 pandemic. Source: Author’s calculation.

To analyse the impact of the COVID-19 pandemic on household income and income inequality, the micro-simulation method is applied to the CHIP 2013 database containing 19,798 urban residents in 6674 households and 38,945 rural residents in 10,490 households. Among the residents, 10,119 urban labourers and 16,483 rural labourers are in off-farm jobs. Based on the percentage changes in earnings of different labourer types simulated by the CHINAGEM model, the changes in household per capita income are calculated. Then, the residents are categorised into 10 groups according to their per capita income. The impact of the COVID-19 pandemic on income inequality could be revealed by comparing the changes in household per capita income by income group (Table 2) and examining the indicators for income inequality, such as the Gini coefficient and Lorenz curve (Figure 7, Figure 8 and Figure 9). 

While the COVID-19 pandemic would significantly reduce urban and rural residents’ household per capita income, residents with lower income levels would suffer larger decreases in their per capita income. On average, the per capita income for the whole nation would fall by 7.87%. Rural residents would experience a more significant decrease in per capita income than urban residents, although the pandemic would cause greater damage to the earnings of urban labourers. This results from the fact that labour income accounts for a higher proportion of rural households’ income. Compared with high-income residents, residents with lower income levels would suffer greater losses in per capita income. The per capita income of residents with the lowest 10% income would decline by 17.58% for the whole nation, 11.84% for urban residents, and 20.11% for rural residents. Comparably, the per capita disposable income of residents with the highest 10% income would decline by 4.78% for the whole nation, 4.81% for urban residents, and 5.70% for rural residents. This result can be explained by two factors. On the one hand, the COVID-19 pandemic would reduce the earnings of labourers with low education levels by a larger amount, and rural residents mostly had lower education levels than urban residents. On the other hand, labour income accounted for a larger proportion in the disposable income of the low-income households. By reducing the per capita income of low-income residents by a larger amount, especially for those living in rural areas, the COVID-19 pandemic would aggravate income inequality in China and threaten the living of poor residents.

As indicated by Lorenz curves and Gini coefficients, the COVID-19 pandemic would aggravate income inequality in China. At the national level, the Gini coefficient would increase from 0.42 before the pandemic to 0.44 affected by the pandemic, accompanied by the Lorenz curve moving down and to the right (Figure 7), which suggests that the COVID-19 pandemic aggravates income inequality in China. The aggravated income inequality of the nation is derived not only from the increased income gap between urban and rural residents but also the income inequality among urban or rural residents. Before the pandemic, urban and rural residents had Gini coefficients of 0.35 and 0.39, respectively, indicating a moderately greater income inequality for the rural residents. As shown in Figure 8 and Figure 9, influenced by the COVID-19 pandemic, the Gini coefficients of urban and rural residents would increase to 0.36 and 0.41, respectively, accompanied by the Lorenz curves moving down and to the right. Hence, for either urban or rural residents, the COVID-19 pandemic would aggravate income inequality among them. This is a result from the fact that the pandemic would reduce household per capita income by a greater amount for residents with a lower income.

The impact of the COVID-19 pandemic is simulated on residents in poverty. The rate of poverty incidence is defined as the ratio of the number of the rural residents that have household per capita income below the poverty line to the number of rural residents. The rate of poverty incidence in China reached 0.60% in 2019 [58]. Although a household sample for 2020 is unavailable, it is still possible to simulate how the COVID-19 pandemic affects the rate of poverty incidence using the CHIP 2013 dataset. To do this, first, a threshold for the poverty line with the sub-samples of rural households is calculated by setting the poverty incidence rate at 0.60%. Then, calculate the number of rural residents with household per capita income below this simulated poverty line, which provides the poverty incidence rate during the pandemic. The results demonstrate that 2.11% of rural residents would have household per capita incomes below the poverty line due to the pandemic. This is also a result of the significant decreases in the per capita income of low-income households. In other words, the pandemic would raise the poverty incidence rate by 1.51 percentage points. However, it is surprisingly observed that China’s government had completely eradicated poverty in 2020, which is contrary to our simulation results. The inconsistency could be explained by the fact that the poverty alleviation policies implemented by China’s governments are not considered after the pandemic outbreak [59].

## 5. Conclusions and Discussions

The COVID-19 pandemic deeply affected China’s macro-economy and labour market, consequently precipitating great shocks to household income. Compared with wealthy households, low-income ones are more vulnerable to the pandemic, as the pandemic results in greater losses to their employment and labour earnings. However, the unequal impact of the COVID-19 pandemic on the labour market and household income in China are poorly tackled by previous studies. Combining a multisector CGE model (CHINAGEM) with a micro-simulation approach, this study develops an illustrative scenario to analyse the unequal impact of the COVID-19 pandemic on the labour market and household income in China. The study contributes to the literature from three perspectives. First, through incorporating a detailed labour matrix into the CHINAGEM model, the unequal impact of the COVID-19 pandemic on the employment and earnings of different labourer types are quantitatively assessed. Second, combining the CGE model with a micro-simulation approach, this study reveals the impact of the COVID-19 pandemic on the per capita income of households with different income levels. Third, to the best of our knowledge, this is the first study to examine how the COVID-19 pandemic affects income equality for rural and urban residents in China. 

The results show that: (1) the COVID-19 pandemic would cause significant damage to China’s macro-economy by reducing China’s national GDP and total employment by 3.77% and 6.72%, respectively. (2) The pandemic would have a far-reaching, heterogeneous impact on the employment and earnings of different labourer types. The labourers who are the female, live in urban areas, and have relatively low education levels would suffer the largest losses in employment and earnings. (3) Affected by the pandemic, per capita incomes would decline by 7.87% for the whole nation, 8.75% for rural households and 6.13% for urban households. While the pandemic would have a larger negative impact on the employment of urban labourers, it would have a greater negative impact on the per capita incomes of rural residents. (4) The per capita income of low-income households is more vulnerable to the pandemic than wealthy households. Per capita income of rural and urban households with the lowest 10% income would decline by 20.11% and 11.84%, respectively. (5) The COVID-19 pandemic would exacerbate income inequality for both rural and urban households and raise the poverty incidence rate by 1.51 percentage points. Overall, the COVID-19 pandemic would aggravate income inequality in China and threaten the living of the poor families, which should be treated with timely and powerful governmental policies. The results verify the research hypothesis that the COVID-19 pandemic would have an unequal impact on labour market and income inequality in China.

In general, the simulation results of this study are fundamentally consistent with those of previous studies. First, for the economic losses, the previous studies projected that China’s GDP in 2020 would decline by 2.71–5% [26,45,46]. This study simulated that China’s GDP in 2020 would fall by 3.77%, which is within this range. Given the IMF’s projection before the pandemic (6%), this study indicates that China’s GDP would grow by 2.23% (=6% − 3.77%) in 2020, which is close to the official statistic (2.30% [4]). Second, this study suggests that total employment would fall by 6.72%, which is close to the projection by Liu et al. [60]. They projected that total employment would decline by 5.96%. However, the reduction in total employment in this study is much higher than in Cui et al. [26] and Zhou et al. [56], as they suggested that total employment would decline by 2.72% and 1.70%, respectively. This disparity could be explained by the fact that these studies employed early data to calculate the pandemic shocks, and consequently under-estimated economic losses. Third, this study found that labourers who are female, live in urban areas, and have low education levels would suffer greater losses in employment and earnings, which is consistent with the findings of previous research [10,11,12,13,14,15]. Fourth, several studies suggested that the COVID-19 pandemic would have a greater negative impact on the per capita income of rural households, compared with their urban counterparts [16,30,36]. This study observed a similar phenomenon, but it initiatively gauged the changes in the income inequality of rural and urban households. Therefore, the results of this study are consistent with those of previous studies, but this study provides more insightful findings on the unequal impact of the COVID-19 pandemic on the labour market and income inequality. 

This study has several limitations. First, the data employed by this study were available in mid-2021, including the data of the 6th nationwide population census of China (surveyed in 2010), the Chinese Household Income Project (CHIP) database (surveyed in 2013), and the national input-output table of 2017. Thus, the data are somewhat outdated, considering the COVID-19 pandemic began in 2020. To alleviate the bias from the utilisation of these data, this study concentrates on the percentage changes in labourer employment and earnings as well as household income rather than their absolute values. Second, although households’ total income collected by the CHIP survey contained the income earned from labourer employment, business activities, government transfers, and other sources, only labour income was reported separately. Hence, it is assumed that the non-labour income of households remains unchanged under the impact of the COVID-19 pandemic. However, the pandemic caused significant damages to business activities, which have a larger negative effect on the income of wealthy households. This fact indicates that the change in income inequality may be overestimated owing to data unavailability. Furthermore, China’s government enforced a series of timely and active policies for low-income households to create jobs and increase their income through government transfers and targeted poverty alleviation programs. As a result, over 5.51 million populations got out of poverty in 2020. Hence, the simulation results of this study without considering governmental poverty alleviation policies may somewhat overestimate the distribution effects of the COVID-19 pandemic, but it could still provide a benchmark for future studies.

This study puts forward three policies to mitigate the adverse impact of the COVID-19 pandemic on China’s labour market and income inequality, which also provide important implications for other developing countries that are hit by the pandemic. First, to maintain labourer employment, more beneficial policies should be given to firms that suffer significant economic losses, particularly those engaged in the real estate, hotels, restaurants, and financial services sectors. To reduce production costs and ease shortages of funds for these firms, the government could extend the tax payment, reduce taxes, relax loan restrictions, and give them low-interest loans. Second, the government should identify the labourers most vulnerable to the COVID-19 pandemic, such as labourers who are the female, urban residents, and less-educated. Unemployed labourers should be given more supportive policies, such as the provision of professional training, living subsidies, and information on job opportunities to improve their labour skills and maintain their living. Moreover, the government could hire less-skilled, less-educated, and rural labourers who live below the poverty line in public service positions, such as street cleaners, river administrators, and road guards. Third, to continuously reduce income inequality, the government should not only improve social welfare policies, such as expanding the coverage of urban unemployment insurance to rural migrant workers, but also raise income transfers and reform the income redistribution mechanisms [61,62]. China’s government has implemented the policies related to the first two policy implications. Although the third implication has not resulted in specific policies, it should be considered strongly by the government to narrow the income gap and achieve the goal of common prosperity. Our research also provides important implications for other developing countries hit by the pandemic.

## Figures and Tables

**Table 1:** The impact of the COVID-19 pandemic on employment and earnings by labourer type (%).

Region	Gender	Education	Employment	Earnings	Region	Gender	Education	Employment	Earnings
Urban	Male	E1	−7.00	−7.26	Rural	Male	E1	−6.02	−3.79
		E2	−7.48	−8.02			E2	−6.39	−5.33
		E3	−7.14	−7.46			E3	−6.49	−6.00
		E4	−6.02	−5.94			E4	−5.70	−5.51
		E5	−5.47	−5.54			E5	−5.53	−5.45
		E6	−5.25	−6.22			E6	−6.03	−6.14
	Female	E1	−8.00	−8.16		Female	E1	−6.51	−3.74
		E2	−8.57	−9.45			E2	−7.36	−5.91
		E3	−7.62	−8.11			E3	−6.86	−6.02
		E4	−5.72	−5.82			E4	−5.71	−5.67
		E5	−5.56	−5.94			E5	−5.40	−5.60
		E6	−5.32	−6.40			E6	−6.01	−6.21

E1–E6 represent elementary school, middle school, high school, junior college, regular college, and postgraduate, respectively. Source: CHINAGEM model.

**Table 2:** Percentage changes in household per capita income of residents with different income levels (%).

Income Groups	Nation	Urban Residents	Rural Residents
[0%, 10%)	−17.48	−11.84	−20.11
[10%, 20%)	−9.79	−8.14	−10.31
[20%, 30%)	−7.94	−6.39	−9.24
[30%, 40%)	−7.01	−6.21	−7.51
[40%, 50%)	−7.45	−5.39	−7.01
[50%, 60%)	−6.76	−5.12	−6.93
[60%, 70%)	−6.56	−4.50	−7.23
[70%, 80%)	−5.78	−4.37	−6.91
[80%, 90%)	−5.12	−4.55	−6.50
[90%, 100%]	−4.78	−4.81	−5.70
Average	−7.87	−6.13	−8.75

Source: Author’s calculation.

## Data Availability

The 6th Chinese Population Census database is available on the website of the National Bureau of Statistics of China: http://www.stats.gov.cn/ztjc/zdtjgz/zgrkpc/dlcrkpc/dlcrkpczl/index.html (accessed on 1 January 2022). The CHIP 2013 database is available in China Institute of Income Distribution, Beijing Normal University: http://www.ciidbnu.org/chip/ (accessed on 1 January 2022); the Input–output table 2017 of China is available on the website of the National Bureau of Statistics of China: https://data.stats.gov.cn/ifnormal.htm?u=/files/html/quickSearch/trcc/trcc01.html&h=740 (accessed on 1 January 2022).

## Abbreviations

COVID-19	Coronavirus disease 2019
CGE model	Computable general equilibrium model
GDP	Gross domestic product
CES	Constant elasticity of substitution
CPC 2010	6th Chinese Population Census
CHIP 2013	Chinese Household Income Project 2013
IMF	International Monetary Fund
CPI	Consumer price index

## Appendix A

**Table A1:** The abbreviations of 19 aggregated sectors.

No.	Abbreviation	Aggregated Sectors
1	AFF	Agriculture
2	MIN	Mining
3	MAN	Manufacturing
4	EGW	Energy and water supply
5	CST	Construction
6	WHR	Trade
7	TWP	Transportation, storage, and post
8	ACC	Accommodation and food services
9	TSI	Information and technology services
10	FIN	Finance
11	REE	Real estate
12	LCS	Leasing and business services
13	STG	Scientific research and development
14	WEP	Water and environment administration
15	RES	Residential services
16	EDU	Education
17	HSW	Health and social welfare
18	CSE	Culture and entertainment
19	SSP	Public administration

Source: The authors.

**Table A2:** The sectorial concordance between the aggregated sectors and the original sectors in the input–output table of 2017.

No.	Original Sectors	Aggregated Sectors	No.	Original Sectors	Aggregated Sectors
1	Farming	AFF	76	Special-purpose machinery	MAN
2	Forestry	AFF	77	Vehicles	MAN
3	Livestock	AFF	78	Vehicle parts and accessories	MAN
4	Fishery	AFF	79	Railway transport equipment	MAN
5	Agricultural services	AFF	80	Boats and ships	MAN
6	Coal mining	MIN	81	Other transport equipment	MAN
7	Crude petroleum and natural gas	MIN	82	Generators	MAN
8	Ferrous metal ores mining	MIN	83	Power transmission equipment	MAN
9	Non-ferrous metal ores mining	MIN	84	Wire and electrical goods	MAN
10	Nonmetallic mineral mining	MIN	85	Batteries	MAN
11	Mining services	MIN	86	Household appliances	MAN
12	Grain processing	MAN	87	Other electrical equipment	MAN
13	Feed processing	MAN	88	Computer	MAN
14	Vegetable oil processing	MAN	89	Communication equipment	MAN
15	Sugar processing	MAN	90	Broadcasting and television equipment	MAN
16	Meat processing	MAN	91	Audiovisual apparatus	MAN
17	Aquatic processing	MAN	92	Electronic parts	MAN
18	Other food processing	MAN	93	Other electronic equipment	MAN
19	Convenience food products	MAN	94	Measuring instruments	MAN
20	Dairy products	MAN	95	Other manufacture	MAN
21	Flavouring products	MAN	96	Waste recycling	MAN
22	Other foods	MAN	97	Machinery and equipment repair	MAN
23	Alcohol	MAN	98	Electricity and steam supply	EGW
24	Soft drinks	MAN	99	Gas supply	EGW
25	Tea	MAN	100	Water supply	EGW
26	Tobacco	MAN	101	Construction of buildings	CST
27	Cotton and chemical fibre spinning	MAN	102	Civil engineering	CST
28	Wool spinning	MAN	103	Construction installation	CST
29	Silk fibre spinning	MAN	104	Construction decoration	CST
30	Knitted fabrics	MAN	105	Wholesale	WHR
31	Textile products	MAN	106	Retail	WHR
32	Textile wearing apparel	MAN	107	Railway passenger transport	TWP
33	Leather products	MAN	108	Railway freight transport	TWP
34	Footwear	MAN	109	Road passenger transport	TWP
35	Timber processing	MAN	110	Road freight transport	TWP
36	Furniture	MAN	111	Water passenger transport	TWP
37	Paper	MAN	112	Water cargo transport	TWP
38	Printing	MAN	113	Air passenger transport	TWP
39	Art and craft product	MAN	114	Air cargo transport	TWP
40	Culture and sport goods	MAN	115	Pipeline transport	TWP
41	Petroleum Products	MAN	116	Transport services	TWP
42	Coking	MAN	117	Storage	TWP
43	Basic chemicals	MAN	118	Post	TWP
44	Fertilizers	MAN	119	Accommodation	ACC
45	Pesticides	MAN	120	Food and Beverage Services	ACC
46	Paints	MAN	121	Telecommunication	TSI
47	Synthetic materials	MAN	122	Radio and television services	TSI
48	Special chemical products	MAN	123	Internet services	TSI
49	Daily-use chemical products	MAN	124	Software services	TSI
50	Pharmaceutical products	MAN	125	Information technology services	TSI
51	Chemical fibres	MAN	126	Financial services	FIN
52	Rubber products	MAN	127	Capital market services	FIN
53	Plastic products	MAN	128	Insurance	FIN
54	Cement and plaster	MAN	129	Real estate	REE
55	Plaster and cement products	MAN	130	Renting and leasing	LCS
56	Building materials	MAN	131	Business services	LCS
57	Glass	MAN	132	Research and development	STG
58	Porcelain products	MAN	133	Professional technique services	STG
59	Refractory products	MAN	134	Technique promotion services	STG
60	Nonmetallic mineral products	MAN	135	Water management	WEP
61	Steel casting	MAN	136	Environmental management	WEP
62	Steel products	MAN	137	Public facilities management	WEP
63	Iron products	MAN	138	Residential services	RES
64	Non-ferrous metal casting	MAN	139	Other services	RES
65	Non-ferrous metal products	MAN	140	Education	EDU
66	Metal products	MAN	141	Health care	HSW
67	Boiler	MAN	142	Social work	HSW
68	Metalworking machinery	MAN	143	Journalism and publishing	CSE
69	Lifting and handling equipment	MAN	144	Televisions and movies	CSE
70	Pump, valve, and compressor	MAN	145	Culture, art, and entertainment	CSE
71	Culture and office equipment	MAN	146	Sports	CSE
72	General-purpose machinery	MAN	147	Recreation	CSE
73	Mining and metallurgy machinery	MAN	148	Social Security	SSP
74	Chemical industry machinery	MAN	149	Public management	SSP
75	Agriculture machinery	MAN			

Source: The authors.
